# Supplementary material for: Hairy Fluorescent Nanospheres Based on Polyelectrolyte Brush for Highly Sensitive Determination of Cu(II)
Source: Polymers (Basel). 2020 Mar 5;12(3):577. doi: 10.3390/polym12030577 (PMC7182828; doi:10.3390/polym12030577)
Supplement: Supplementary file 1 [file polymers-12-00577-s001.pdf]

Article

Hairy Fluorescent Nanospheres Based on Polyelectrolyte Brush for Highly Sensitive Determination of Cu(II)

Qiaoling Wang [1], Kaimin Chen [1,*], Yi Qu [1,*], Kai Li [1], Ying Zhang [2] and Enyu Fu [1]

[1] College of Chemistry and Chemical Engineering, Shanghai University of Engineering Science, Shanghai 201620, China; woodsues@outlook.com (Q.W.); 18301939658@163.com (K.L.); fuenyu1234@163.com (E.F.)

[2] School of Chemical Engineering, East China University of Science and Technology, Shanghai 200237, China; zy12fearless@163.com

* Correspondence: kmchen@sues.edu.cn (K.C.); quyi@fudan.edu.cn (Y.Q.)

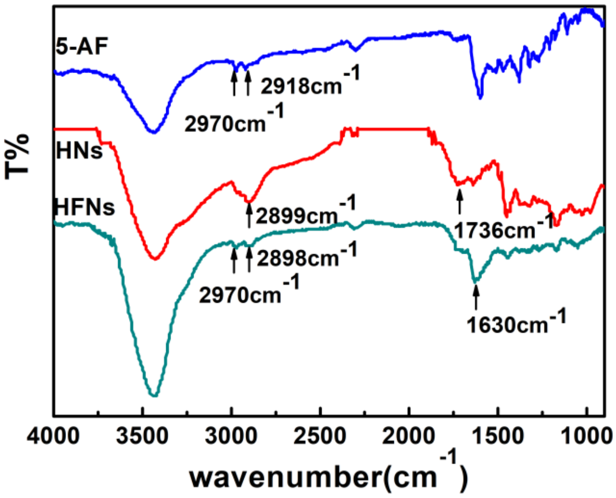

Figure S1. FTIR absorption spectra of 5-AF, hairy nanospheres and hairy fluorescent nanospheres

Surface functionalization of HNs and 5-AF were characterized with Fourier transform infrared spectroscopy (As shown in the figure s1). For 5-aminofluorescein, there is O-H stretching vibration at 3600-3100 $cm^{-1}$and C=C exists at 2000-1650 cm^{-1} with broad frequency absorption. For HNs, the C=O group is at 1736cm^{-1} and the obvious hydroxyl polymerization signal exists at 3200-2500 cm^{-1}. The energy band near 1630 cm^{-1} corresponds to the bending of the amine NH, the stretching vibration of NH at 3650-3200 cm^{-1} and the internal vibration of the amide bond at about 1380 cm^{-1}, which indicates successful coupling for 5-AF.
